# The Chinese Mandarin Version of the Esophageal-Atresia-Quality-of-Life Questionnaires for Children and Adolescents: Evaluation of Linguistic and Content Validity

**DOI:** 10.3390/ijerph192214923

**Published:** 2022-11-13

**Authors:** Siqi Li, Michaela Dellenmark-Blom, Yong Zhao, Yichao Gu, Shuangshuang Li, Shen Yang, Julia H. Quitmann, Jinshi Huang

**Affiliations:** 1 Department of Neonatal Surgery, Beijing Children’s Hospital, Capital Medical University, National Center for Children’s Health, Beijing 100045, China; 2 Department of Pediatric Surgery, Queen Silvia Children’s Hospital, Sahlgrenska University Hospital, 416 85 Gothenburg, Sweden; 3 Department of Pediatrics, Institute of Clinical Sciences, Gothenburg University, The Queen Silvia Children’s Hospital, 416 86 Gothenburg, Sweden; 4 Department of Medical Psychology, University Medical Center Hamburg-Eppendorf, Martinistraße 52, D-20246 Hamburg, Germany; 5 Department of Neonatal Surgery, The Affiliated Children’s Hospital of Nanchang University, Nanchang 330006, China

**Keywords:** esophageal atresia, quality of life, cognitive debriefing, cross-cultural translation, content validity

## Abstract

Background: After repair of esophageal atresia (EA), children risk digestive and respiratory morbidity, but knowledge of their health-related quality of life (HRQOL) in China is lacking. The EA-QOL questionnaires were developed in Sweden and Germany to evaluate condition-specific HRQOL in children with EA aged 2–7 and 8–17. This study aimed to evaluate the linguistic and content validity of the Chinese Mandarin version of the EA-QOL questionnaires. Methods: The procedure was conducted in compliance with international standards, including a forward-backward translation procedure, expert reviews, and cognitive debriefing interviews with 14 Chinese families of children with EA (parents of 8 children aged 2–7/6 children aged 8–17 and their parents). Results: Following forward-backward translation, minor issues were identified and solved. In interviews, all participants rated all EA-QOL items easy to understand, none expressed negative emotions about them and most described them comprehensive and relevant for EA. Leading from cognitive debriefing, three EA-QOL items in the questionnaire version for children aged 2–7 and three EA-QOL items in the questionnaire version for children aged 8–17 were modified in the Chinese language to improve cultural appropriateness and/or clarity. Conclusion: The Chinese Mandarin version of the EA-QOL questionnaires achieved satisfactory linguistic and content validity. This can help increase focus of HRQOL in research and clinical practice of children with EA in China.

## 1. Introduction

Esophageal atresia (EA) refers to a discontinuity of the esophagus and has an incidence of 1/2500–1/4500 [1]. In recent years, the survival rates after repair of EA have increased and have reached 93–95% [2,3]. Although the operation has restored the continuity of esophageal anatomy, children often face digestive and respiratory morbidity, growth retardation and mental health disorders [1]. Therefore, after repair of EA, patients may experience both physical and mental health problems in daily life. A study from China found that the reasons for treatment abandonment in children with EA included financial difficulties, poor prognosis, postoperative complications, and extensive length of intensive care unit stay in the past [4]. However, following improvement of the economic level among the citizens in China, and advancement in the management of children with EA, more children have successfully survived. Increased attention has therefore been paid to provide adequate follow-up care to monitor the child and family health.

According to the World Health Organization, the concept Quality of Life (QOL) refers to an individual’s perception of one’s position in life in the context of the culture and value systems in which they live, and in relation to their goals, expectations, standards and concerns. QOL is multidimensional and incorporates a person’s subjective experience of his/her physical, psychological and social state [5]. The term Health-related quality of life (HRQOL) regards a person’s perception of the impact of health and treatment on physical, psychological, and social functioning and well-being [6]. The scoping review from Witt et al. showed the importance to use both the children’s self-reports and their parent proxy reports to understand HRQOL in pediatric patients [6]. Condition-specific questionnaires capture HRQOL aspects of relevance to people with a particular disease/condition and can provide clinically important information to clinicians [7]. A standardized condition-specific HRQOL questionnaire with sound validity and reliability in multiple countries is needed to increase statistical power in HRQOL evaluations of people living with a rare condition [8]. According to international recommendations of patient-reported outcome measures, children’s and parents’ views are of outmost importance to the development and evaluation of a HRQOL instrument, in order to achieve an adequate instrument design for the specific patient group, age and culture [9].

In children with EA, HRQOL research have expanded the recent years; however, no study of children with EA living in China has been reported [10,11,12]. In previous studies, the use of generic HRQOL instruments have shown both worse HRQOL [13,14,15] and similar HRQOL [16] in children with EA compared to healthy references. Recently, a set of condition-specific HRQOL questionnaires for children with EA aged 2–7 and 8–17 years was developed and evaluated for feasibility, validity and reliability in Sweden and Germany [17,18,19]. Then, studies have demonstrated overall acceptable psychometric performance of the EA-QOL questionnaires among children with EA in Turkey, Poland and the Netherlands [20,21,22]. Many QOL/HRQOL questionnaires have been translated from English to Chinese, and the Chinese version of the questionnaires has achieved satisfactory validity and reliability [23,24,25,26,27,28,29]. In order to increase knowledge of HRQOL of children with EA in China, it is central to investigate the psychometric properties of the EA-QOL questionnaires among Chinese families of children with EA. In this study, we report the translation, initial cultural adaptation, and content validity of the Chinese Mandarin version of the EA-QOL questionnaires, when used in with families of EA living in China, prior to completing a field test.

## 2. Materials and Methods

### 2.1. The EA-QOL Questionnaires

**Figure 1:**
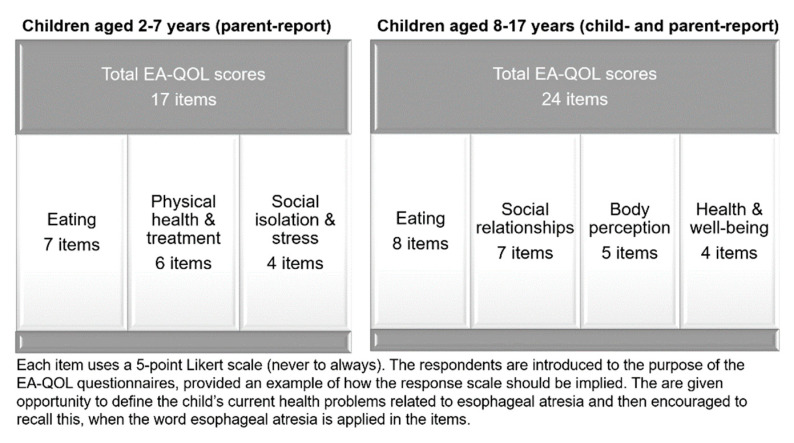
Format of the EA-QOL questionnaires for children aged 2–7 and 8–17.

In congruence with international standards of patient-reported outcome measurement, the EA-QOL questionnaires were developed based on condition-specific HRQOL experiences reported in focus groups with children and their parents from Sweden [19]. The reported HRQOL experiences were used to generate items for the EA-QOL questionnaires. The items underwent an item reduction process and evaluation through a pilot- and a field test in families of children with EA from Sweden and Germany [17,18]. The structure and content of the EA-QOL questionnaires are illustrated in Figure 1.

### 2.2. Framework

The translation process was guided by the International Society of Pharmacoeconomics and Outcome Research (ISPOR) Principles of Good Practice for the Translation and Cultural Adaptation Process for Patient-Reported Outcomes Measures [30], including the phases (1) Preparation (2) Forward Translation (3) Reconciliation (4) Back Translation (5) Back Translation Review (6) Harmonization (7) Cognitive Debriefing (8) Review of Cognitive Debriefing Results (9) Finalization and (10) Proofreading and Final Report.

### 2.3. Preparation—Step 1

After signing the License and User Agreement of translation and validation of the EA-QOL questionnaires, the key-in country person (SL) was provided a study protocol in English. In agreement with the abovementioned recommendations by ISPOR principles [30], the study protocol was developed to aid conceptual equivalence of the different translations of the EA-QOL questionnaires and standardize the psychometric evaluation across countries. Therefore, the study protocol detailed the procedure of translation, cognitive debriefing and the field test. The study protocol included a description of each item, item key words and presented quotes from children and parents from the Swedish focus groups [19]. The instrument developer from Sweden (MDB) and key-in-country person from China (SL) worked as main contact persons throughout the study. A representative from Germany (JHQ), who participated in the development of the EA-QOL questionnaires was available for consultation when needed.

### 2.4. Translation—Step 2–6

**Figure 2:**
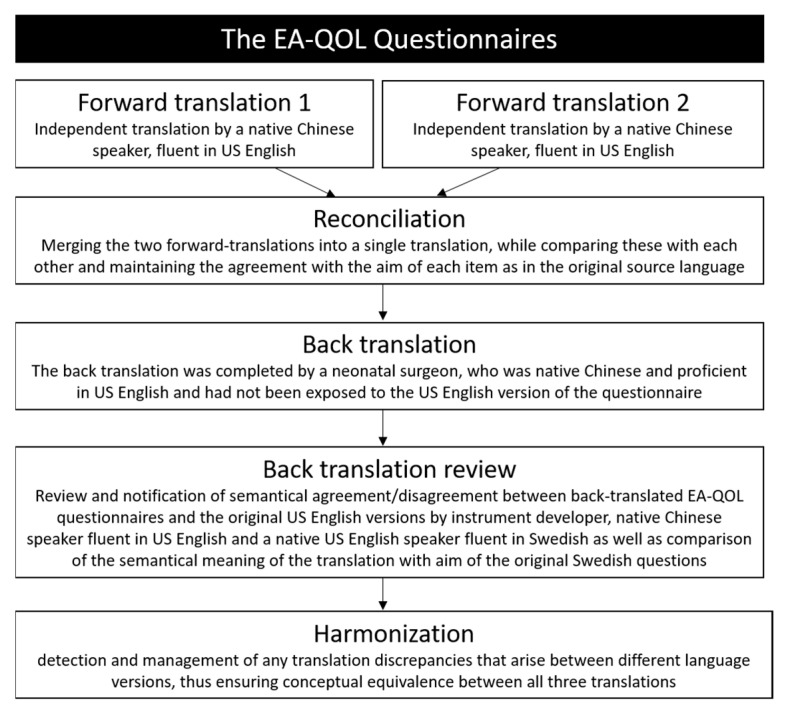
Translation procedure.

The process of translation and harmonization is outlined in Figure 2. Since Sweden is a low-population country, we encountered difficulties recruiting native Chinese-Swedish translators and therefore the US English version of the EA-QOL questionnaires was used as a mediating translation to reach conceptual equivalence between the Chinese Mandarin translation and the original Swedish source language. The two forward translators were native speakers of the target language Chinese Mandarin, resided in the target country China, were fluent in English as having qualified through the Chinese College English test 6, which has been recognized by foreign universities and they had experience in the translation of questionnaires. When two independent forward translations of the EA-QOL questionnaires from US English into Chinese Mandarin were finalized, these were reviewed by the key-in-country person, who was also native speaker of Chinese Mandarin, resident in the target country, had a medical health background and was fluent in English. The key-in-country person noted semantical agreement/disagreement between the two forward-translations and the forward translations with the item information from the study protocol in order to determine the reconciliation, and issues that appeared were discussed with a translator or instrument developer when needed. The reconciled Chinese Mandarin translation of the EA-QOL questionnaires underwent a back translation into US English by a translator who had not been exposed to the US English version of the questionnaires. In the back-translation review, the semantical agreement/disagreements between the back-translation and the US English version of the EA-QOL questionnaires were noted by MDB, then reviewed by the key-in country person (SL), a native US English speaker fluent in Swedish (outside the research team) and the instrument developer who was native Swedish speaker and fluent in English language (MDB). If any result indicated a need to revise the Chinese Mandarin translation of the EA-QOL questionnaires, it was discussed in the research group, until consensus of a good Chinese Mandarin translation was reached. In this way, the Chinese Mandarin translation of the EA-QOL questionnaires was harmonized with the US English and Swedish source language (Figure 2).

### 2.5. Cognitive Debriefing—Step 7

Cognitive debriefing is a standard procedure to perform interviews with respondents from the target population of an instrument, in order to test and provide evidence of its content validity. It refers to a research technique which aims to identify and resolve unclarity or inadequacy in wording or cultural appropriateness of the translations, thereby optimizing the children’s and parents’ understanding of the items [31]. We used convenience sampling and recruited families of children with EA aged 2–17 years for the cognitive debriefing interviews from two hospitals in China between June 2020 and December 2020. The study sample was stratified for severity of EA as presented in Table 1 to ensure that children with different severity of disease and their parent-proxies were interviewed. All participants were native Chinese Mandarin speakers and had no cognitive impairment and could read fluently.

The cognitive debriefing interviews were conducted by a researcher with experience of communicating with pediatric patients and their parents, but who was not personally involved in the treatment of children with EA. The cognitive debriefing interviews were conducted one-on-one through video calls, and hence the interviews with children aged 8–17 years and their parents were conducted separately. Approximately one week prior to the interview, participants received an informed consent form (ICF) and a copy of the questionnaire through WeChat—a communication service application [32]. They were asked to read, sign, and return the ICF in advance of the interview. At the start of the interview, the interviewer provided a brief description of the purpose of the interview. After having obtained the participant’s consent, electronic records were made at the same time as the interview. As a start, the interview briefly focused on collecting information of the child’s past treatment history and current health state, and then continued to the cognitive debriefing of the EA-QOL questionnaires. The interviewer encouraged the child/parent to read the questionnaire’s instruction and then to reply to the items. For each item, the interviewer asked him/her if the item was easy to understand, and if not, what made the item unclear. The interviewer also asked if the item was sensitive to answer, and if so, what was sensitive. Furthermore, the informants were encouraged to share ideas for item improvements from their perspective. After each questionnaire domain (e.g., Eating) the interviewer encouraged the child/parent to describe if the items of the domain were comprehensive to a child/adolescent with EA from their point of view. The interviewer made careful field notes during the interview, including the participants feedback of the questionnaire instructions, response scale and relevance of the questions. The participants were then sent a standardized questionnaire to collect basic clinical information.

### 2.6. Review of Cognitive Debriefing Results—Step 8

Content validity refers to evidence that the questionnaire instructions, items, domains and response scale are appropriate relative to their intended measurement concept, population and use [31,33]. Therefore, the field noted experiences reported by children and/or parents were translated into English. Using a deductive content analysis, the child- and parent- reported experiences of each item were categorized into affirmative statements and statements regarding item ambiguity. The categorization process was conducted by two authors (SL, MDB) and finalized when consensus was reached. The combination of participant rating and comments of the EA-QOL items served as information to the author team, whether these were valid for content, needed cultural adaptation and/or improvement in wording for clarity.

### 2.7. Finalization—Step 9

The Chinese Mandarin translation of the EA-QOL questionnaires was reviewed by the key-in-country person (SL) to check for accuracy compared to the US English version of the EA-QOL questionnaires.

### 2.8. Proof-Reading and Final Results—Step 10

The finalized Chinese Mandarin translation of the EA-QOL questionnaires was proofread by the Chinese research team to check for minor errors, which could have been missed during the translation process. For this final report, the author team aimed to explain the methodology of use, reasons for all translation/wording choices and decisions undertaken throughout the translation process.

## 3. Results

### 3.1. Translation—Step 2–6

Two forward translators completed the translation of the questionnaire from US English to Chinese Mandarin language independently. In the process of reconciliation, a few minor discrepancies were detected and solved. For example, there was a difference in the translation of “a full meal”, whether it regarded a large (大份) or a normal-sized (正常量) meal. Following reconciliation, it was translated into a normal-sized meal. Moreover, the word “complicated (复杂)” was translated into “difficult (困难) “, but was changed to the first mentioned concept, consistent with the original wording.

Table 2 gives an overview of the backtranslation and backtranslation review. As shown, the backtranslation had overall good conceptual agreement with US English version of the EA-QOL questionnaire. A total of 10 items were revised after author review and discussions. Additionally, issues detected in the translational procedure regarded the word “choke”, which could be translated in two ways in Chinese Mandarin language and therefore these translations were remained to consult respondents in the cognitive debriefing for appropriateness.

### 3.2. Cognitive Debriefing—Step 7

A total of 14 families of children who had been surgically treated for EA participated in the cognitive debriefing. The study sample included 8 children aged 2–7 years (male: 6, female: 2), four of whom were considered to have mild, two moderate and two severe EA. Moreover, 6 children aged 8–17 years (male: 5, female: 1), three were represented mild and three moderate EA. Table 3 summarizes the further characteristics of EA patients and their families.

### 3.3. Review of Cognitive Debriefing Results—Step 8

All participants rated all questions of the EA-QOL questionnaires for children aged 2–7 years and 8–17 years as clear and easy to understand, and none expressed negative emotions about any of the issues or felt that the items were sensitive to answer. According to the participants, the questionnaires were adequate and targeted for EA and covered content of all possible problems children with EA may encounter. No complaints were made on the questionnaire instructions or response scale.

The qualitative results attained through the cognitive debriefing with parents of children aged 2–7, and with children aged 8–17 and their parents are presented in Supplemental Material S1. The table presentations in Supplemental Material S1 include comments indicating affirmation and ambiguity of the items and the research team’s decision whether the item wording needed improvement or not. As shown, there were affirmative experiences of all items in the EA-QOL questionnaire for children aged 2–7 and in all but one item (no 14) in the EA-QOL questionnaire for children aged 8–17. Nevertheless, comments by respondents also indicated that eight items in the EA-QOL questionnaire for children aged 2–7 and seven items in the EA-QOL questionnaire for children aged 8–17 needed discussion regarding item comprehensibility. In each age-specific questionnaire, three items were decided to be culturally adapted to improve item clarity, as explained below:

- One item of the EA-QOL questionnaire for children with EA aged 2–7 (no 1, regarding impact on the child’s eating due to food sticking in the throat) and for children aged 8–17 years, respectively (no 1, regarding impact on the child’s eating due to food getting stuck in the throat), both include the expression “food gets stuck in the throat”, which were literally translated as “食物卡在喉咙里” in Chinese Mandarin language. However, most participants stated that food often gets stuck in the esophagus, not the throat. Since “food gets stuck in the throat” was an idiomatic expression, the final Chinese Mandarin translation was adjusted to “食物卡在食管里”.
- The term “choke” has two meanings in Chinese Mandarin language, namely “food gets stuck in the esophagus and compresses the airway, preventing the child from breathing normally (窒息)” and “cough caused by inhalation of food into the trachea while eating (呛咳)”. The word “choke” is used in one item of the EA-QOL questionnaire for children aged 2–7 years (no 5, regarding the child’s experiences of worry when choking on food) and two items of the EA-QOL questionnaire for children aged 8–17 years (no 5 regarding the child’s feelings of fear when choking during eating, and no 6 regarding the child’s difficulties to eat a meal due to choking experiences). Since most participants recognized the second situation and this maintained the conceptual equivalence, we retained this wording.
- In one item of the EA-QOL questionnaire for children aged 2–7 years (no 7, regarding limitations of social activities and events which include eating with peers), parents are asked about their child’s experience of having problems to eat food at a party or when he/she is out with friends. Few parents in the cognitive debriefing would let their child go to parties or eat with friends, because their child’s food needs to be prepared by themselves. This would make it unfeasible for parents of children who have not dined outside home to reply to this item. It was therefore adjusted in wording to increase clarity in a Chinese setting, to ask about problems for the child to attend parties or being out with friends due to eating difficulties. The final Chinese Mandarin translation was adjusted to “您的孩子参加聚会或与朋友外出吃饭时是否有问题”.

### 3.4. Finalization—Step 9

The Chinese translation of the EA-QOL questionnaires reached accuracy in the final comparison to the US English version of the EA-QOL questionnaires.

### 3.5. Final Review and Final Report—Step 10

The finalized Chinese translation of the EA-QOL questionnaires were successfully checked for minor errors which could have been missed during the translation. The final Mandarin translations of the EA-QOL questionnaires for children aged 2–7 and 8–17 years are provided in Supplemental Material S2.

## 4. Discussion

This study described the translation, initial cultural adaptation, and content validity of the Chinese Mandarin version of the EA-QOL questionnaires for children and adolescents, prior to commencing our field test study. To the authors knowledge it is the first report of HRQOL in pediatric patients with EA from China, obtaining both children’s and parent’s perspectives.

In China, we urgently need an indicator to understand the outcomes of survivors after repair of EA. Moreover, a HRQOL questionnaire could help guiding postoperative monitoring of the child’s health in follow-up care and family support [1]. The need for a standardized condition-specific HRQOL questionnaire for children with EA to be used across countries has been addressed [17]. At present, the EA-QOL questionnaires have demonstrated overall acceptable validity and reliability for use in Sweden, Germany, Turkey Poland and the Netherlands [17,20,21,22].

In this study, the forward-backward translation of the EA-QOL questionnaires showed good results and minor issues were easily solved. Moreover, in line with the ISPOR principles, the procedure also identified one issue carried on to the cognitive debriefing in order to await the respondent’s view on the accuracy of the Chinese Mandarin translation. In order to achieve accurate, cross-culturally and conceptually equivalent versions of the EA-QOL questionnaires, formal methods of translation and adaptation need to be applied. Such international recommendations of translation are available to standardize the terminology, procedure and prevail poorly translated instruments that threaten the validity of research data, including in global data sets and meta-analysis of the measured HRQOL outcomes [34]. However, rare conditions pose challenges to the translation of a HRQOL questionnaire, why the standard procedure may need practical adaptation, such as producing less forward-backward translations [35,36]. Moreover, many HRQOL measures have been developed in English [35], and have been translated from English into Chinese [23,24,25,26,27,28,29]. The EA-QOL questionnaires were developed in the Swedish language, and Sweden is a low-population country. This added further challenges to this study and contributed to difficulties recruiting Swedish-Chinese Mandarin translators. Nevertheless, when translating and validating a HRQOL questionnaire for children with a rare disease, collaboration is one of the cornerstones. As we did not want to hamper HRQOL research of children with EA living in China and the possible future contribution to international multi-center studies on the topic, we agreed to use the established US English version of the EA-QOL questionnaires, to mediate the translation into Chinese Mandarin language and to use a bilingual back translator who was not native in US English, which is a clear study weakness. At the same time, we decided to optimize a good Chinese Mandarin translation through performing two independent forward translations, having a key-in-country consultant as the main contact person managing the process in the target country (SL) who was a native speaker of the target language Chinese Mandarin, fluent in the source language English, resident in the target country China and had a medical health profession. We also used a careful description of aim, keywords and patient/parent reports each of the EA-QOL item in the study protocol and a thorough discussion between representatives in the target country and the instrument developers (MDB, JQ). Moreover, we included a native US English reviewer of the backtranslation. Together the involved participants, bridged all languages used in the translational procedure.

Following translation, the cognitive debriefing of the Chinese Mandarin version of the EA-QOL questionnaire was conducted in agreement with international recommendations; it focused on all relevant aspects of the instrument including the instructions, items, concepts, vocabulary and in age group 8–17 years, children were interviewed regarding the self-report and parents regarding the parent-proxy report of the EA-QOL questionnaires. Furthermore, the cognitive debriefing reached the recommended sample size in the literature [9,30,37]. In the initial Swedish–German evaluation of the EA-QOL questionnaires, severe EA was defined as one or more of the following criteria; primary anastomosis was delayed and/or esophageal replacement was performed, major revisional surgery due to anastomotic leakage or recurrent fistula, severe tracheomalacia/tracheobronchomalacia verified through most recent broncoscopy, presence of associated anomaly resulting in disability according to the definition provided by the World Health Organization [19]. The stratification of severity of EA used in this study was different. The explanation is that although the definition of severe EA used in the Swedish-German evaluation was comprehensive, it would require that study centers provide long-term follow-up care for patients with EA and have access to detailed historical medical record data, which is not internationally applicable. By providing guidance for stratification for severity of EA in the study protocol based on information mainly collected at the study assessment, all new countries translating and validating the EA-QOL questionnaires can use a standardized stratification method for severity of the disease in the cognitive debriefing.

Based on the results in the cognitive debriefing, the Chinese version of the EA-QOL questionnaire achieved content validity, as all items were considered understandable, relevant and comprehensive. Most of the participants were open and enjoyed helping with their experiences. However, the cognitive debriefing also identified three issues within the eating domain. Two concepts were adjusted to the appropriate linguistic use in Chinese Mandarin language and one item was reworded to better suit the Chinese family behaviors. Moreover, the cognitive debriefing shed light on aspects which currently did not require cultural adaptation, but still will be important when interpreting the future field test results. This regarded the implied social behaviors of people in China, including family communication patterns in children aged 8–17. For example, children’s feelings of it being complicated to explain EA to other people may be related to how they are exposed to other people’s questions and to how much the children are educated by parents or healthcare professionals about their condition. We found that some parents revealed the real situation to their child very briefly and that they and their children chose not to discuss EA with other people. Furthermore, some parents did not want to tell their children about EA and declined study participation. This is in line with Matza et al., suggesting that there may be cultural differences in the information that is revealed to children about a disease, and variations in the level to which children are regarded as self-reliant reporters of their HRQOL [8]. However, application of HRQOL measures in a clinical setting with children, have contributed to providing them insights about their health [38] and increased communication of HRQOL issues with the clinician [39]. Moreover, the typical activities among children may differ across countries [9], and we found that some children in China will try to avoid exposing their scars in public, by covering their scars with clothes. This could cause difficulties to respond to an item about the situation when their scar(s) become visible to other people. However, this item was well understood by the children, rated as not sensitive to answer, and the interviewed children were relatively young with restricted social lives. Moreover, the participants considered that impact of scar(s) on the child’s HRQOL was well addressed in the EA-QOL questionnaire.

The study sample achieved the size and was stratified to ensure that children with different severity of EA was represented in the cognitive debriefing in accordance with suggestions made in previous literature, Still, this study is limited by use of convenient sampling, with participants recruited from populations attending follow-up at two hospitals in China, and by the fact that several families declined study participation. The variation in the children’s access to follow-up care after repair of EA in China, different socioeconomic conditions among the families and communication patterns of among the affected families to speak with their child about EA represents aspects that could influence children’s perception of their HRQOL. This illustrates the complexity of HRQOL research and should be considered in relation to our study findings. Moreover, this study was limited to focus on the Chinese Mandarin language, and not Chinese Cantonese language, which is also spoken among citizens in China. Additionally, when a HRQOL questionnaire for children is evaluated in a new country, consideration of possible differences in educational system between countries are recommended, because it could impact the understanding of HRQOL items [9]. In the current study, only two children in age group 2–7 had started preschool. This may influence the parents’ perception of items asking about their children’s social situation due to EA. Moreover, most of the parent proxy report were mothers. However, this is commonly observed in HRQOL studies of children [40], and the parent caring for their child may provide most reliable information about the child’s HRQOL. This study has focused on the evaluation of linguistic and content validity of the EA-QOL questionnaires in Chinese Mandarin language. Conceptually, there seem to be important similarities in condition-specific HRQOL issues across countries, including China. Such homogeneity in the definition of condition-specific HRQOL does however not imply that participants ratings of the HRQOL are similar across countries. Future research is therefore needed to investigate if the EA-QOL questionnaires measure the latent construct equivalently in all country/cultural groups under investigation.

## 5. Conclusions

The Chinese Mandarin version of the EA-QOL questionnaires for children aged 2–7 (parent report) and children aged 8–17 (child and parent report) achieved sound linguistic and content validity. As perceived by the participating families of children in China, it represents a targeted tool for HRQOL of children and adolescents with EA. Following, further psychometric evaluation of the Chinese Mandarin version of the EA-QOL questionnaires in a field test, these questionnaires can be made available for research of children with EA This study can in the long-term contribute with an increased focus of HRQOL research and clinical practice of children with EA living in China.

## Figures and Tables

**Table 1:** Severity of EA.

	Clinical Characteristics of the Patient
Severe	Clinically significant dysphagia
	Clinically significant gastro-esophageal reflux disease
	Received dilatation of esophagus
	Airway disease
	Clinical significant associated anomaly
Moderate	Clinically significant dysphagia, gastro-esophageal reflux disease, received dilatation of esophagus
	OR
	Clinically significant airway disease
	With associated anomaly
Mild	Dysphagia OR gastroesophageal reflux disease OR airway disease
	No associated anomaly

**Table 2:** An overview of the backtranslation review, including comparison of the back-translation from Chinese Mandarin into US English, the original US English version and the Swedish version of the EA-QOL questionnaires for children with esophageal atresia.

The EA-QOL Questionnaires	Total Number of Items	Number of Items with Conceptual Agreement	Number of Items with Conceptual Inconsistences	Need for Minor Revisions in the Chinese Translation after Discussion in the Research Team	Minor Revisions in the Chinese Translation
**Children aged 2–7 (parent report)**					
Eating	7	2	5	4	“difficulty swallowing food” was changed to “difficulty to eat” “a regular meal” was changed to “a full meal” Removed extra examples in two items
Physical health & treatment	6	5	1	1	“high respiratory infections”—“high” was omitted
Social isolation & stress	4	3	1	1	Item wording of school absence modified
**Children aged 8–17 (child report/parent report)**					
Eating	8	6	2	0	
Social relationship	7	6	1	1	Do you “think” was corrected to “feel” in an item asking about feelings about the child feeling like the only one with esophageal atresia
Body perception	5	5	0	0	
Health and well-being	4	3	1	1	Item wording was shortened from, esophageal atresia makes me “sad or upset” to using only “sad”

**Table 3:** Presentation of families responding to the EA-QOL questionnaires for children aged 2–7 and for children aged 8–17 (n = 6).

	Children Aged 2–7 (n = 8)	Children Aged 8–17 Years (n = 6)
**Child background information**		
Male (n, %)	6 (66.7)	5 (83.3)
Gestational age (median and P25/P75, weeks)	37.2 (37.0, 38.2)	37 (37.0, 39.7)
Birth weight (median and P25/P75, grams)	2600 (2275, 3003)	3100 (2375, 3200)
Primary esophageal repair (n, %)	7 (87.5)	5 (83.3)
Revisional surgery (n, %)	5 (62.5)	1 (16.7)
Associated anomalies (n, %)	3 (37.5)	2 (33.3)
Child age (median and P25/P75, year)	3 (2.3, 4.5)	9.5 (8, 11.5)
**Parental information**		
Mother (n, %)	8 (100.0)	3 (50.0)
Parental age (median and P25/P75, year)	33.0 (33.0, 36.0)	39.5 (37.8, 46.5)
Cohabitant partner (n, %)	8 (100.0)	6 (100.0)
College/University educated (n, %)	4 (50.0)	2 (33.3)

## Data Availability

The data presented in this study are available on request from the corresponding author.

## Supplementary Materials

The following supporting information can be downloaded at: https://www.mdpi.com/article/10.3390/ijerph192214923/s1, S1: Cognitive debriefing results of the Chinese Mandarin version of the EA-QOL questionnaires for children with esophageal atresia, S2: The Chinese Mandarin version of the EA-QOL questionnaires for children aged 2–7 (parent report) and children aged 8–17 (child- and parent- report).
