# Supplementary material for: The Chinese Mandarin Version of the Esophageal-Atresia-Quality-of-Life Questionnaires for Children and Adolescents: Evaluation of Linguistic and Content Validity
Source: Int J Environ Res Public Health. 2022 Nov 13;19(22):14923. doi: 10.3390/ijerph192214923 (PMC9690468; doi:10.3390/ijerph192214923)
Supplement: Supplementary file 1 [file ijerph-19-14923-s001.zip › Supplementary material S1.pdf]

Supplemental material 1a. Cognitive debriefing results of the Chinese Mandarin version of the EA-QOL questionnaire for children with esophageal atresia aged 2-7 years (parent-proxy-report).

Domain		Content of the item	Ambiguity?	Ambiguity, statement	Affirmative	Affirmative statement, example	Change/modification of item
		Assess the impact on the child's…					
Eating	1.	eating due to food sticking in the throat	Yes, regarding food gets stuck "in the throat" vs "the esophagus"	Food sometimes gets stuck in the esophagus stenosis, rather than the throat	Yes	Food is often stuck and even noodles	Yes, adjusted to "食物卡在食管里"
	2.	ability to eat a full meal	Yes, one statement	Item will be clearer if there was an example given	Yes	My child can't eat normal food because of his narrow esophagus, so he can only chew the food to feel the taste and then spit it out	No, remain the original structure
	3.	eating-related stress	No		Yes	My child likes to eat liquid food because he is afraid that he may feel uncomfortable when eating	No
	4.	satisfaction with their own eating pace	No		Yes	My child eats slowly, but always wants to eat	No
	5.	worry when choking on food	Yes, there are two ways to translate "choke" in Chinese		Yes	Sometimes my child will cough when eating fast, but it does not affect his breathing	Yes, translation consistent with choking as cough caused by inhalation of food into the trachea while eating (呛咳) and with the original EA-QOL item
	6.	experience of vomiting	No		Yes	Eating large pieces of food can cause vomiting	
	7.	limitations regarding social activities and events that include eating with peers	Yes, for the children not attending or children needing adjustement to attend social activities that include eating	While we were worried about the child's problem with eating, we (parents) basically didn't take him out to eat	Yes	When we go to the party, we have to bring the child her own food	Yes, adjusted to ask for problems attending parties or being out with friends due to their eating (您的孩子参加聚会或与朋友外出吃饭时是否有问题)
**Physical Health & Well-being**	8.	experience of physical tiredness when playing games or sports due to their condition	Yes	Similarity between question 8 and 9	Yes	My child tires easily when he exercises	No, these questions regard different aspects of the child's physical health and children having both of these problems will probably have lower condition-

	9.	ability to perform physically demanding activities in daily life	Yes	Similarity between question 8 and 9	Yes	My child has less strength than other children	specific health-related quality of life. Moreover, it was the cross-cultural structure of the questionnaire
	10.	experiences of being bothered by respiratory symptoms	No		Yes	My child was short of breath and wheezing during exercise, so he felt different from other children	No
	11.	experience of having problems with getting respiratory infections easily	No		Yes	My child often has respiratory tract infection, so sometimes he will worry about why he is different from other children	No
	12.	negative emotions regarding the need for taking medicines	Yes, one statement regarding the content	All children hate taking medicine	Yes	My child hates medicine very much	No, item performance is overall good
	13.	ability to fall or stay asleep at night due to their health condition	No		Yes	My child can't sleep well at night because of reflux	No
**Social Isolation & Stress**	14.	negative life experiences because of preschool/school absence	Yes, regarding how to answer the question if the child has not started school	My child hasn't started school yet	Yes	My child was absent from kindergarten classes because of frequent illness	No, remain the cross-cultural structure of the questionnaire
	15.	social stress due to the need for explaining their condition/abilities to other people	Yes, regarding the child's lacking ability to understand the situation	My child is not aware of this yet	Yes	My child will patiently explain these questions to others	No, remain the cross-cultural structure of the questionnaire
	16.	experiences of social stigma/social stress due to people's comments about them	No		Yes	When my child has comments, he doesn't worry about them	No
	17.	experiences of social exclusion/stress due to people's negative reactions of the child making a noise	Yes, regarding the child's lacking ability to understand of the situation	My child is not aware of this yet	Yes	My child's voice is hoarse when crying, which other parents find it strange	No, remain the cross-cultural structure of the questionnaire

Supplemental material 1b. Cognitive debriefing results of the Chinese Mandarin version of the EA-QOL questionnaires for children with esophageal atresia aged 8-17 years (self- and parent-proxy-report).

Domain		Core content of the item	Ambiguity	Ambiguity, statement	Affirmative	Affirmative statement, example	Led to change/modification of item?
		Assess impact on the child's…					
**Eating**	1.	eating due to food getting stuck in the throat	Yes	Food will get stuck in the anastomosis of his esophagus, not in his throat (parent)	Yes	Food often gets stuck (child)	Yes, adjusted to "食物卡在食管里"
	2.	need for restricting his/her eating of certain foods'	No		Yes	I can't eat big pieces of food (child)	
	3.	experience of pain when eating	No		Yes	I feel pain when food gets stuck in my esophagus or gastroesophageal reflux (child)	
	4.	experience of the need to consciously think of drinking a lot (remind themselves) when eating	No		Yes	I will remind myself to drink water when eating to prevent food from getting stuck in the esophagus (child)	
	5.	feelings of fear when choking during eating	Yes	Recently, food stuck in the esophagus did not make me unable to breathe, but it sometimes made me cough (child)	Yes	He sometimes eats fast which causes him cough	Yes, translation consistent with choking as cough caused by inhalation of food into the trachea while eating (呛咳) and with the original EA-QOL item
	6.	difficulties to eat a meal due to choking experiences	Yes	One statement, that question 5 and question 6 are similar (parent)	Yes	I don't think it's difficult to eat. I just cough when I eat too fast (child-report)	Yes, translation consistent with choking as cough caused by inhalation of food into the trachea while eating (呛咳) and with the original EA-QOL item
	7.	ability to eat at the same pace as peers	No		Yes	I'm used to eating slower than others (child)	
	8.	experience of vomiting after meals	No		Yes	Regurgitation of stomach contents into his mouth will bother him (parent)	
**Social Relationships**	9.	experience of emotional isolation like being the only one with their condition	No		Yes	He feels he is the only child with esophageal atresia (parent)	
	10.	experience that it is complicated to explain their condition to others	Yes	No one asked me what esophageal atresia is (child)	Yes	He can explain esophageal atresia in one sentence	No, overall good item performance, therefore, remain the original item wording
	11.	experience of being called names by others due to their condition	No		Yes	In rural areas, superstitious old people will say something bad	

	12.	perception that others stare at you because of their condition	No		Yes	He is afraid that others will see his scar, so he will change clothes (parent)	
	13.	experience of being tired of the need to explain their scar(s) to other people	Yes	Nobody asked me this question (child)	Yes	He hates people asking him questions about scars, but he will answer patiently (parent)	No, overall good item performance, therefore, remain the original item wording
	14.	perception of others saying unkind things about them	No		No		
	15.	emotions due to other people’s questions about their condition	No		Yes	My child won't find it hard to deal with, but he will be embarrassed (parent)	
Body Perception	16.	experience of feeling different due to surgical scar(s)	No		Yes	My child cares about his scars, but does not feel much different from others (parent)	
	17.	concern regarding what to wear because of the surgical scar(s)	No		Yes	My child only wears clothes that can cover the scar (parent)	
	18.	unease related to the scar(s) being visible to others	Yes	I won't let anyone see my scars	Yes	When I swim, people can see my scar, I will feel embarrassed (child)	Since the children who were interviewed were relatively young and few seemed to have collective lives, and qualitative finds showed mixed results, this item was retained
	19.	satisfaction of scar(s) on their looks	No		Yes	My child thinks his scars as uncomely (parent)	
	20.	experience of being small for age	Yes	I'm not shorter than my peers (child)	Yes	I'm thinner than my peers, but it doesn't bother me (child)	No, since it is known that esophageal atresia is a heterogenous condition
Health & Well-being	21.	Experience of breathing difficulties when they exercise and play	No		Yes	He has obvious shortness of breath when he exercises, which affects his physical education results (parent)	
	22.	ability to fall or stay asleep at night due to their condition	No		Yes	Sometimes gastroesophageal reflux makes me feel heartburn, which can cause me sleeping difficulties (child)	
	23.	worry about the future due their condition	No		Yes	My child is worried that the scar will affect his job search (child)	

24.	feelings of being sad due to their condition	No	Yes	The first time my parents told me that I had surgery for esophageal atresia after I was born, I felt very sad, but now I don't feel sad (child)
