# Supplementary material for: The Chinese Mandarin Version of the Esophageal-Atresia-Quality-of-Life Questionnaires for Children and Adolescents: Evaluation of Linguistic and Content Validity
Source: Int J Environ Res Public Health. 2022 Nov 13;19(22):14923. doi: 10.3390/ijerph192214923 (PMC9690468; doi:10.3390/ijerph192214923)
Supplement: Supplementary file 1 [file ijerph-19-14923-s001.zip › Supplementary material S2.pdf]

编号：____________

日期：____________

Quality-of-life questions for Young Children born with Esophageal Atresia

食管闭锁患儿生活质量调查问卷（2-7 岁）

父母报告

您好！我们想通过问卷了解您的孩子的健康状况和感觉如何？

这些问题都是食管闭锁患儿术后可能经常遇到的，是由其他食管闭锁患儿和他们的家长提出的。

您的孩子生后因食管闭锁（食管不连续，伴或不伴食管与气管相通）进行手术治疗。术后您的孩子可能会有一些症状，请勾选。

- ☐ 食物卡在他/她的食管里
- ☐ 反酸或烧心
- ☐ 呛咳
- ☐ 气道问题，例如咳嗽，粘痰，呼吸困难，喘息，哮喘
- ☐ 他/她身材比同龄人矮小
- ☐ 他/她需要药物治疗
- ☐ 他/她还有其他疾病影响他/她的健康
- ☐ 其他____________________________

当提到食管闭锁时，我们希望您可以考虑到您的孩子存在的所有问题。

- *请您根据<u>过去 4 个星期</u>的情况来回答以下问题。*
- *这些问题没有正确答案！您只需要根据自己孩子的情况如实回答。*
- *如果您不理解该问题或不想回答该问题，请跳过该问题，然后尝试下一个。*

例如：

您的孩子听他/她喜欢的音乐…

想想<u>过去的 4 个星期</u>

	从不	很少	有时	经常	总是
他/她听他/她喜欢的音乐					

Copyright © 2017. Michaela Dellenmark- Blom and Kate Abrahamsson, Queen Silvia Children's Hospital, Gothenburg Sweden. The federation of esophageal atresia and tracheo-esophageal fistula support groups e.v., EAT, board [patient representatives]. All rights reserved.
Copyright © 2022. 黄金狮，首都医科大学附属北京儿童医院新生儿外科。All rights reserved.

想想<u>过去的 4 个星期</u>

关于您的孩子的饮食情况	从不	很少	有时	经常	总是
1、您的孩子是否由于食物会卡在他/她的食管里而感到吃东西困难？					
2、您的孩子吃饱饭会有困难吗？					
3、您的孩子有吃东西相关的压力吗？					
4、您的孩子可以按照他/她想要的速度吃饭吗？					
5、您的孩子吃饭时会担心自己会呛咳吗？					
6、呕吐是否对您的孩子造成了困扰？					
7、您的孩子参加聚会或与朋友外出吃饭时是否有问题？					

关于您的孩子的身体健康和治疗	从不	很少	有时	经常	总是
8、您的孩子玩游戏或运动时容易疲倦吗？					
9、在体力活动中，您孩子的体力是否比其他孩子差？					
10、您的孩子是否会因为呼吸道问题感到困扰（例如咳嗽，有痰或呼吸困难）？					
11、您的孩子容易发生呼吸道感染吗？					
12、您的孩子讨厌吃药吗？					
13、您的孩子的健康情况是否会使他/她晚上睡得不好？					

关于您的孩子与他人的相处	从不	很少	有时	经常	总是
14、您的孩子是否会由于健康问题而导致缺课（学前班/小学），缺课的频率是怎样的?					

Copyright © 2017. Michaela Dellenmark- Blom and Kate Abrahamsson, Queen Silvia Children's Hospital, Gothenburg Sweden. The federation of esophageal atresia and tracheo-esophageal fistula support groups e.v., EAT, board [patient representatives]. All rights reserved.
Copyright © 2022. 黄金狮，首都医科大学附属北京儿童医院新生儿外科。All rights reserved.

15、您的孩子很难向他人解释他/她可以做什么和不能做什么?					
16、别人对您的孩子的反应/评论是否会使他/她感到烦恼?					
17、您的孩子会因为自己发出的声音惊吓到他人而感到困扰吗（例如：跑步时的呼吸声、清嗓声、咳嗽声）？					

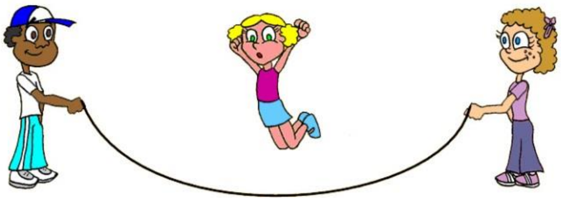

在这里您可以写一些我们没有问到的情况

....

....

....

....

....

非常感谢您的回答！

Copyright © 2017. Michaela Dellenmark- Blom and Kate Abrahamsson, Queen Silvia Children's Hospital, Gothenburg Sweden. The federation of esophageal atresia and tracheo-esophageal fistula support groups e.v., EAT, board [patient representatives]. All rights reserved.
Copyright © 2022. 黄金狮，首都医科大学附属北京儿童医院新生儿外科。All rights reserved.

编号：______________

日期：______________

Quality of Life questionnaire for children and adolescents born with esophageal atresia

食管闭锁患儿生活质量调查问卷（8-17 岁）

父母报告

您好！我们想通过问卷了解您的孩子的健康状况和感觉如何？

这些问题都是食管闭锁患儿术后可能经常遇到的，是由其他食管闭锁患儿和他们的家长提出的。

您的孩子生后因食管闭锁（食管不连续，伴或不伴食管与气管相通）进行手术治疗。术后您的孩子可能会有一些症状，请勾选。

- ☐ 食物卡在他/她的食管里
- ☐ 反酸或烧心
- ☐ 呛咳
- ☐ 气道问题，例如咳嗽，粘痰，呼吸困难，喘息，哮喘
- ☐ 他/她身材比同龄人矮小
- ☐ 他/她需要药物治疗
- ☐ 他/她还有其他疾病影响他/她的健康
- ☐ 其他____

当提到食管闭锁时，我们希望您可以考虑到您的孩子存在的所有问题。

- *请您根据过去 4 个星期的情况来回答以下问题。*
- *这些问题没有正确答案！您只需要根据自己孩子的情况如实回答。*
- *如果您不理解该问题或不想回答该问题，请跳过该问题，然后尝试下一个。*

例如：

您的孩子听他/她喜欢的音乐…

*想想**过去的 4 个星期***

	从不	很少	有时	经常	总是
我的孩子听他/她喜欢的音乐					

Copyright © 2017. Michaela Dellenmark- Blom and Kate Abrahamsson, Queen Silvia Children's Hospital, Gothenburg Sweden. The federation of esophageal atresia and tracheo-esophageal fistula support groups e.v., EAT, board [patient representatives]. All rights reserved.
Copyright © 2022. 黄金狮，首都医科大学附属北京儿童医院新生儿外科。All rights reserved.

想想<u>过去的 4 个星期</u>

关于您的孩子的饮食情况	从不	很少	有时	经常	总是
1、吃饭时食物卡在食管里会使您的孩子感到困扰吗？					
2、您的孩子的健康状况是否会限制他/她吃某些食物？					
3、您的孩子吃饭的时候会因为一些情况感到疼痛吗（例如：食物卡在喉咙里，烧心，胃痛）？					
4、您的孩子吃饭的时候会反复提醒自己要多喝水吗？					
5、您的孩子呛咳的时候会害怕吗？					
6、有时会呛咳会使您的孩子觉得吃东西很难吗?					
7、您的孩子可以和其他同龄的孩子以相同的速度吃饭吗？					
8、吃完饭后呕吐是否对您的孩子造成了困扰？					

关于您的孩子和他人相处	从不	很少	有时	经常	总是
9、您的孩子是否觉得他/她是唯一的食管闭锁的孩子？					
10、您的孩子觉得向其他人解释食管闭锁很复杂吗？					
11、有人给您的孩子起外号吗（也许是因为身材矮小，咳嗽，进食缓慢或手术疤痕）？					
12、您的孩子是否感觉有人在盯着他/她（例如：他/她咳嗽时，呛咳时，在更衣室换衣服时）？					
13、您的孩子是否厌烦别人询问有关他/她的疤痕的问题？					
14、有人对您的孩子说一些不好听的话吗？					

Copyright © 2017. Michaela Dellenmark- Blom and Kate Abrahamsson, Queen Silvia Children's Hospital, Gothenburg Sweden. The federation of esophageal atresia and tracheo-esophageal fistula support groups e.v., EAT, board [patient representatives]. All rights reserved.
Copyright © 2022. 黄金狮，首都医科大学附属北京儿童医院新生儿外科。All rights reserved.

15、当其他人向您的孩子询问食管闭锁时，他/她会感到难对付吗？					

关于身体和瘢痕	从不	很少	有时	经常	总是
16、您的孩子是否因为有疤痕而觉得自己和其他人不一样？					
17、您的孩子是否因为疤痕在意自己的穿着？					
18、当其他人（例如：新朋友，男/女朋友，更衣室/游泳池里的人）看到他/她的疤痕时，他/她是否感到难堪？					
19、您的孩子是否因为疤痕而觉得自己不完美？					
20、您的孩子是否因自己身材比同龄人矮小而感到困扰？					

关于您孩子的健康和幸福	从不	很少	有时	经常	总是
21、如果您的孩子可以运动和玩耍，呼吸困难会给他/她带来麻烦吗？					
22、您的孩子是否因为健康状况（例如：反酸，烧心，呼吸道问题）而在晚上睡不好？					
23、您的孩子是否因为食管闭锁担心自己的未来（例如：学校，朋友，男/女朋友，工作）？					
24、食管闭锁会让您的孩子难过吗？					

在这里您可以写一些我们没有问到的情况

……………………………………………………………………………………………………

非常感谢您的回答！

Copyright © 2017. Michaela Dellenmark- Blom and Kate Abrahamsson, Queen Silvia Children's Hospital, Gothenburg Sweden. The federation of esophageal atresia and tracheo-esophageal fistula support groups e.v., EAT, board [patient representatives]. All rights reserved.
Copyright © 2022. 黄金狮，首都医科大学附属北京儿童医院新生儿外科。All rights reserved.

编号: ______________

日期: ______________

Quality of Life questionnaire for children and adolescents born with esophageal atresia

食管闭锁患儿生活质量调查问卷（8-17 岁）

自我报告

你好！你感觉如何？这是我们想了解的。

这些问题都是食管闭锁患儿术后可能经常遇到的，是由其他食管闭锁患儿和他们的家长提出的。

你出生后因为食管闭锁（食管不连续，伴或不伴食管与气管相通）进行了手术治疗。手术会留下疤痕，你也会有一些其他症状。下面请根据你自己的情况勾选。

- ☐ 食物卡在食管里
- ☐ 反酸或烧心
- ☐ 呛咳
- ☐ 气道问题，例如咳嗽，粘痰，呼吸困难，喘息，哮喘
- ☐ 我身材比同龄人矮小
- ☐ 我需要药物治疗
- ☐ 还有其它疾病影响我的健康
- ☐ 其他____

当提到食管闭锁时，我们希望你能认真考虑自己存在的所有问题。

- *请你根据过去 4 个星期的情况来回答问题。*
- *这些问题没有正确答案！你只需要根据自己的情况如实回答。*
- *如果你不理解该问题或不想回答该问题，请跳过该问题，然后尝试下一个。*

例如：

你听自己喜欢的音乐…

想想过去的 4 个星期

	从不	很少	有时	经常	总是
我听自己喜欢的音乐					

Copyright © 2017. Michaela Dellenmark- Blom and Kate Abrahamsson, Queen Silvia Children's Hospital, Gothenburg Sweden. The federation of esophageal atresia and tracheo-esophageal fistula support groups e.v., EAT, board [patient representatives]. All rights reserved.
Copyright © 2022. 黄金狮，首都医科大学附属北京儿童医院新生儿外科。All rights reserved.

想想<u>过去的 4 个星期</u>

关于饮食	从不	很少	有时	经常	总是
1、吃饭时食物卡在食管里会使你感到困扰吗?					
2、你的身体情况是否会让你不能吃某些食物?					
3、你吃饭的时候会因为一些情况而感到疼痛吗（例如：食物卡在喉咙里，烧心，胃痛）？					
4、你吃饭的时候会反复提醒自己要多喝水吗?					
5、你呛咳的时候会害怕吗?					
6、有有时会呛咳会使你觉得吃东西很难吗?					
7、你吃饭的速度和同龄人相同吗?					
8、吃完饭后呕吐是否对你造成了困扰?					

关于你和他人的相处	从不	很少	有时	经常	总是
9、你是否觉得自己是唯一的食管闭锁的孩子?					
10、你觉得向其他人解释食管闭锁很复杂吗?					
11、有人给你起外号吗（也许是因为身材矮小，咳嗽，进食缓慢或身上的疤痕）？					
12、你是否感觉有人在盯着你（例如：咳嗽时，呛咳时，在更衣室换衣服时）？					
13、你是否厌烦人们问你疤痕的问题？					
14、有人对你说一些不好听的话吗?					
15、当其他人问你关于食管闭锁的问题时，你会感到难对付吗?					

关于身体和瘢痕	从不	很少	有时	经常	总是
16、你是否因为有疤痕而感觉自己和其他人不一样？					

Copyright © 2017. Michaela Dellenmark- Blom and Kate Abrahamsson, Queen Silvia Children's Hospital, Gothenburg Sweden. The federation of esophageal atresia and tracheo-esophageal fistula support groups e.v., EAT, board [patient representatives]. All rights reserved.
Copyright © 2022. 黄金狮，首都医科大学附属北京儿童医院新生儿外科。All rights reserved.

17、你是否因为疤痕在意自己的穿着?					
18、当其他人（例如：新朋友，男/女朋友，更衣室/游泳池里的人）看到你的疤痕时，你是否感到难堪?					
19、你是否因为自己有疤痕而觉得自己不完美?					
20、你是否因为身材比同龄人矮小而感到困扰?					

关于你的健康和幸福	从不	很少	有时	经常	总是
21、如果你可以运动或玩耍，呼吸困难会给你带来麻烦吗?					
22、你的身体情况是否会让你在晚上睡不好（例如：反酸，烧心，呼吸道问题）？					
23、你是否因为食管闭锁担心自己的未来？（例如：学校，朋友，男/女朋友，工作）					
24、食管闭锁会让你难过吗？					

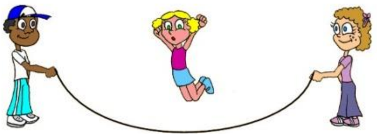

在这里你可以写一些我们没有问到的情况

………………………………………………………………………………………………

………………………………………………………………………………………………

………………………………………………………………………………………………

非常感谢你的回答！

Copyright © 2017. Michaela Dellenmark- Blom and Kate Abrahamsson, Queen Silvia Children's Hospital, Gothenburg Sweden. The federation of esophageal atresia and tracheo-esophageal fistula support groups e.v., EAT, board [patient representatives]. All rights reserved.
Copyright © 2022. 黄金狮，首都医科大学附属北京儿童医院新生儿外科。All rights reserved.
